# High-Pressure Inelastic Neutron Spectroscopy: Experimental Validation of Machine-Learned Interatomic Potential Energy Landscapes

**DOI:** 10.1021/acs.jpclett.6c00720

**Published:** 2026-06-05

**Authors:** Jeff Armstrong, Adam Jackson, Alin Elena

**Affiliations:** † ISIS Neutron and Muon Source, Science and Technology Facilities Council, UK Research and Innovation, Rutherford Appleton Laboratory, Harwell Campus, Didcot, OX11 0QX, U.K.; ‡ Department of Chemistry, University of Bath, Claverton Down, Bath, BA2 7AY, U.K.; § Scientific Computing Department, Science and Technology Facilities Council, UK Research and Innovation, Rutherford Appleton Laboratory, Harwell Campus, Didcot, OX11 0QX, U.K.; ∥ Scientific Computing Department, Science and Technology Facilities Council, UK Research and Innovation, Daresbury Laboratory, Keckwick Lane, Daresbury, WA4 4AD, U.K.

## Abstract

Machine-learned interatomic potentials (MLIPs) promise near density-functional theory accuracy at a fraction of the computational cost, offering a route toward predictive atomistic modeling of molecular and condensed-phase materials. Yet their reliability beyond the training regime remains difficult to establish experimentally. Here we use pressure-dependent broadband inelastic neutron spectroscopy (INS) as an experimental probe of MLIP transferability. Using a low-background NiCrAl high-pressure clamp cell, we measure INS spectra of crystalline 2,5-diiodothiophene at 10 K under atmospheric pressure and at 1.5 GPa. MACE-based MLIPs, fine-tuned on targeted DFT data, reproduce the experimental spectra across 0–1200 cm^–1^ at both pressures and remain stable in finite-temperature molecular dynamics simulations at 300 K. The models capture systematic pressure-induced blue shifts arising from steric stiffening and reproduce an anomalous red shift near 453 cm^–1^ associated with pressure-modified intermolecular interactions. These results demonstrate that pressure-dependent INS can validate how an MLIP responds to a controlled thermodynamic perturbation, testing not only equilibrium structure but also the pressure-dependent curvature of the potential-energy surface. High-pressure INS therefore provides a practical experimental route for validating transferable machine-learned potentials for molecular materials.

## Graphical abstract

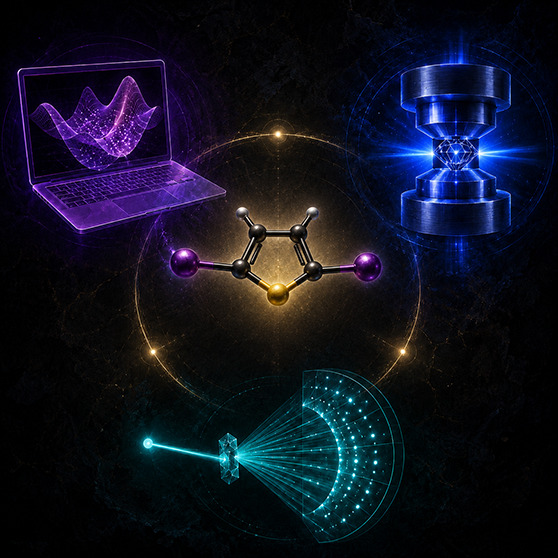

Machine-learned interatomic potentials (MLIPs) have transformed atomistic modeling by delivering accuracy close to density-functional theory (DFT) at a fraction of the computational cost.^1,2^ This enables simulations of vibrational response, collective structural fluctuations, diffusive motion, and slow dynamical processes on spatial and temporal scales directly comparable with experiment. As MLIPs become embedded in predictive materials modeling, however, rigorous experimental validation becomes essential. Crucially, agreement with the first-principles data used for training or with static structural observables^3–5^ does not guarantee that the underlying forces remain accurate when the thermodynamic state is perturbed.

Many technologically relevant materials operate in regimes where weak, cooperative, and environment-dependent interactions dominate their behavior. In zeolites used for catalysis,^6,7^ adsorption and reactivity depend sensitively on collective framework flexibility and subtle many-body interactions between guest molecules and acid sites. In metal–organic frameworks,^8,9^ gas uptake and selectivity emerge from correlated host–guest motion, transient pore distortions, and crowding under confinement. In electrolytes,^10^ ion transport arises from coupled rotational and translational dynamics within fluctuating coordination environments. Organic electronic materials^11–14^ rely on delicate balances between intermolecular packing, low-energy phonons, and dynamic disorder to govern charge mobility. In all such systems, many-body forces govern the accurate description of rotational barriers, diffusion coefficients, vibrational properties, and ultimately the macroscopic transport behavior of the material.

Broadband inelastic neutron spectroscopy (INS) provides a direct experimental probe of these forces because vibrational frequencies correspond to second derivatives of the potential-energy surface, and simulated neutron-weighted spectra can be compared with experiment in a one-to-one manner.^15,16^ Accurate reproduction of INS spectra therefore tests not only equilibrium structure but also the curvature and coupling of the underlying many-body force landscape.

Crucially, a fundamental limitation of INS is that measurements must typically be performed at cryogenic temperatures, constraining temperature as a thermodynamic axis for validating model transferability. This presents a serious challenge, because without demonstrated transferability, the central benefit of MLIPs is eroded, given that their training cost can approach that of ab initio methods. To overcome this central limitation, we recast pressure as a deliberate thermodynamic axis of validation, enabling stringent tests of transferability without sacrificing the sharp spectral resolution of 10 K INS measurements. However, high-pressure INS presents formidable experimental challenges, stemming from the limited sample volumes and significant parasitic scattering associated with bulky pressure cells. Recent high-pressure INS capability on TOSCA results from a combination of instrument and sample-environment developments. The upgrade of the TOSCA primary flight path increased neutron delivery to the sample position, while mechanically robust, low-background NiCrAl pressure cells enabled measurements at gigapascal pressures with reduced parasitic scattering.^17,18^ The present clamp cell belongs to the broader family of NiCrAl, often “Russian alloy”, pressure-cell designs developed for high-pressure INS, including work associated with Lagrange at ILL and the Institute for Nuclear Research RAS, Troitsk.^19^ Its implementation on TOSCA is adapted to the specific detector geometry, sample illumination, and masking requirements of the spectrometer. Together, these developments allow vibrational spectra to be recorded under systematically compressed intermolecular separations within the same crystalline material, probing modified interatomic force constants and providing an experimental test of MLIP transferability under controlled pressure conditions.

As a model system we examine crystalline 2,5-diiodothiophene, whose well-defined INS line shapes and fine dispersion character make it a sensitive probe of subtle changes in the potential-energy surface under compression. As a halogenated thiophene molecular crystal, 2,5-diiodothiophene also provides a chemically relevant model for the thiophene motifs that underpin many organic electronic and polythiophene-based materials.

The measured INS spectra of crystalline 2,5-diiodothiophene at atmospheric pressure and 1.5 GPa retain clearly resolved vibrational features across 0–1200 cm^–1^ following empty-cell subtraction. Representative subtraction spectra are provided in the Supporting Information. The dominant pressure response occurs below 500 cm^–1^ (Figure 1), where multiple bands shift to higher energy under compression, consistent with steric stiffening as intermolecular separations decrease. In contrast, a feature near 453 cm^–1^ exhibits a reproducible red shift. This mode has previously been assigned to an out-of-plane ring deformation coordinate,^20^ demonstrating that compression does not uniformly harden all vibrational degrees of freedom, but can selectively soften specific internal motions through subtle changes in intermolecular coupling.

**Figure 1:**
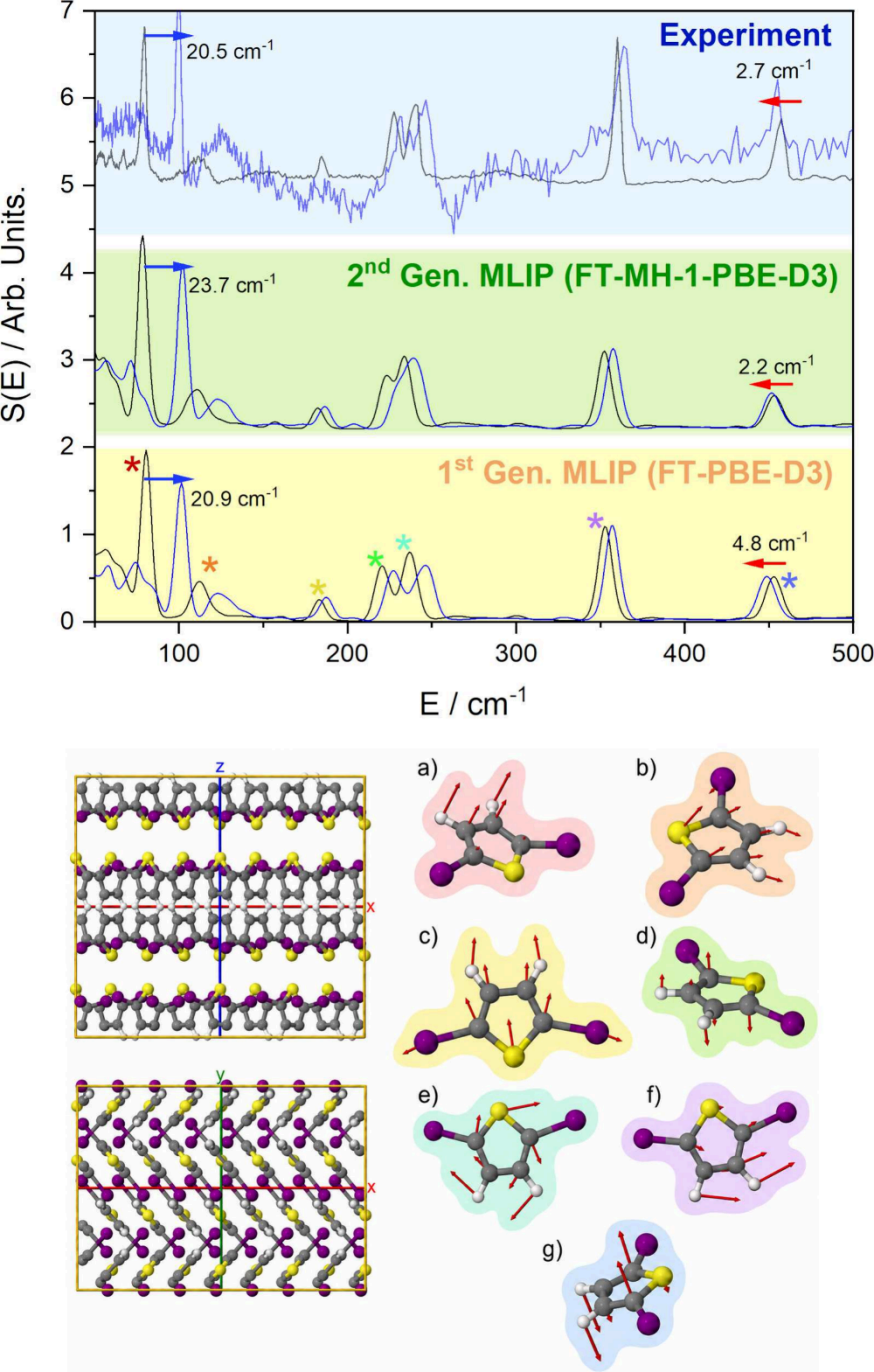
Pressure-dependent INS spectra, structural motif, and representative vibrational mode assignments for crystalline 2,5-diiodothiophene. Top: experimental INS spectra compared with MLIP-derived neutron-weighted spectra for the first- and second-generation fine-tuned models, with black and blue traces denoting atmospheric pressure and 1.5 GPa, respectively. All experimental spectra were collected at 10 K. Blue and red arrows indicate representative pressure-induced blue and red shifts. Colored asterisks in the first-generation MLIP spectrum mark the atmospheric pressure modes assigned in the molecular displacement panels below. Bottom left: MLIP-relaxed packing motif, showing the bilayer structure and herringbone arrangement. Bottom right: a-g show representative molecular displacement patterns across the 80–500 cm^–1^ region, shown in ascending frequency; the translucent colored backgrounds correspond to the colored asterisks in the spectrum. Atom colors are carbon, gray; hydrogen, white; sulfur, yellow; and iodine, purple. Red arrows indicate dominant atomic displacements.

To construct a transferable MLIP suitable for pressure-dependent validation, a first-principles reference description of the potential-energy surface is required. Density functional theory (DFT) calculations were therefore performed using the projector-augmented wave method implemented in VASP to generate an initial PBE-D3­(BJ) training data set.^21–25^ These calculations provided energies, forces, and stresses for pressure-optimized supercells that span both atmospheric pressure and compressed configurations. The system-specific MLIP was then developed by fine-tuning the MACE-MP-0 foundation model on this data.^26^ Rather than relying solely on small harmonic displacements about equilibrium, the training set was iteratively enriched with configurations sampling local strain, anisotropic lattice distortions, equation of state like structures, geometry optimization and finite-temperature fluctuations via molecular dynamics using ASE^27^ and janus-core.^28^ For the exact details of the finetuning configuration selection see the Supporting Information. This refinement strategy exposes the model to regions of configuration space relevant to both thermodynamic states. Such training is particularly demanding for molecular crystals, where weak intermolecular dispersion, steric contacts, and subtle pressure-induced reorientation of the herringbone packing must be captured consistently in order to preserve both structural stability and the correct curvature of the potential-energy surface. Additional models were generated by fine-tuning “second generation” multiheaded MACE foundation models^29^ with the same training configurations and with different density-functional approximations, PBE-D3­(BJ)^23–25^ and R2SCAN-D4.^30–32^ Full details of these models are contained in the Supporting Information.

TOSCA spectra were simulated from the MLIP using Phonopy and AbINS.^15,33^ Figure 1 compares the experimental spectra with the simulated spectra from the first and second generation fine-tuned MLIPs (PBE based). Although the subsequent MLIP phonon calculations are inexpensive, construction of the fine-tuned potential carries a non-negligible upfront cost. In the present case, fine-tuning required hours on A100 GPU resources, while subsequent MLIP geometry optimization and phonon calculations for the 2 × 2 × 4 supercell required only minutes. The computational advantage is therefore not best understood as replacing a single DFT phonon calculation, but as paying the training cost once, then reusing the resulting potential across multiple pressures, supercell sizes, and finite-temperature configurations. Full timing details for the DFT data generation, model training, and MLIP phonon calculations are provided in the Supporting Information. Agreement is near-quantitative across the full measured energy range, with the first and second generation models each performing slightly better in different regions of the spectra. Strikingly, both generations of MLIP reproduce the pressure response of the vibrational modes, including the anomalous red shift at 453 cm^–1^. Because the low-energy region is governed by weak intermolecular interactions and subtle packing rearrangements under compression, accurate reproduction of these pressure-induced shifts demonstrates that the MLIPs correctly describe how the curvature of the intermolecular potential-energy surface evolves with pressure.

To distinguish MLIP fitting accuracy from the quality of the underlying DFT reference, Figure 2 compares the fine-tuned models directly with the PBE-D3­(BJ) vibrational response. Both PBE-based MLIPs reproduce the main DFT neutron-weighted spectral features across the 50–500 cm^–1^ region, including the low-energy modes most sensitive to intermolecular packing. The accompanying phonon-dispersion comparison shows that the first-generation MLIP also captures subtle DFT mode splittings away from the Γ point. This confirms that the pressure-dependent INS agreement in Figure 1 arises from a close MLIP reproduction of the reference potential-energy surface, rather than from agreement at only a small number of isolated peaks.

**Figure 2:**
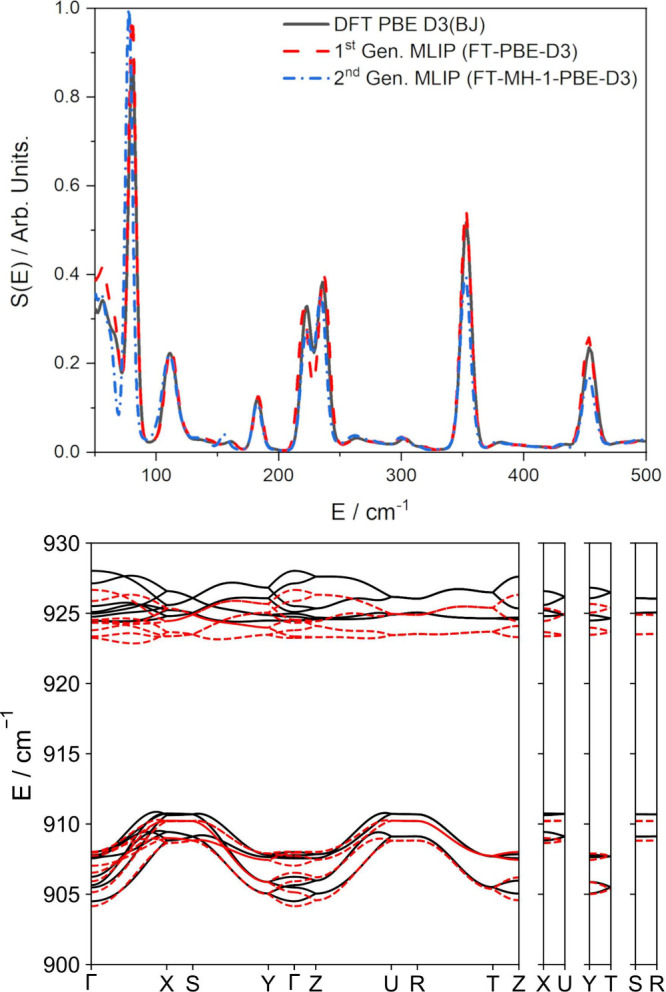
Comparison of DFT and fine-tuned MLIP vibrational predictions at atmospheric pressure. Top: neutron-weighted spectra calculated from the PBE-D3­(BJ) DFT reference and from the first- and second-generation PBE-based MLIPs over the 50–500 cm^–1^ region. The agreement shows that both fine-tuned MLIPs closely reproduce the reference DFT vibrational response in the low-energy region used for the pressure-dependent INS analysis. Bottom: comparison of the DFT and first-generation MLIP phonon dispersions in a narrow high-frequency region, illustrating that the fitted potential also captures subtle dispersion features and mode splittings away from the Γ point.

Analysis of the MLIP-relaxed structures reveals that the crystal comprises halogen-bond-mediated bilayers stacked along the *z*-axis and a distorted herringbone packing motif within the *x*–*y* plane (Figure 1, bottom left panel). Compression to 1.5 GPa produces pronounced anisotropic lattice contraction, with a 4.40% reduction along the *z*-axis (parallel to the halogen-bonded bilayers) and a 2.73% contraction along the *x*-axis. This anisotropy is accommodated by a pressure-induced adjustment of the herringbone tilt angles (Figure 1, bottom left panel), preserving halogen contacts while modifying intermolecular separations and relative molecular orientation. The relative orientation of neighboring rings is altered by 3° under compression from 0 GPa to 1.5 GPa.

The vibrational mode visualizations in Figure 1 (bottom right panel) clarify how these structural changes translate into spectral shifts. The modes that blue shift under compression (Figure 1 a-f) are dominated by collective displacements of the rigid ring motif relative to neighboring molecules. In these cases, the internal geometry of the five-membered ring remains essentially intact; the motion primarily alters intermolecular contact distances. Under compression, such displacements increase steric repulsion between adjacent motifs, steepening the local energy curvature and naturally producing systematic blue shifts. By contrast, the 453 cm^–1^ mode (Figure 1 g) corresponds to an internal out-of-plane deformation of the ring framework itself. Through the tilting adjustment to the herringbone packing, the associated ring–ring electronic overlap changes, reducing the effective restoring force along this coordinate and providing a structural explanation for the observed red shift.

Although the ability to rapidly compute phonon spectra, and therefore any macroscopic properties derived from them, across a wide range of pressures is already highly useful, the ultimate value of an MLIP lies in its ability to describe thermally activated motion and collective behavior of functional materials at finite temperature. Ion migration in solid electrolytes, molecular diffusion in porous frameworks, lattice thermal transport, mechanical response, and thermodynamic observables all emerge from finite-temperature sampling of the underlying energy landscape. A model that reproduces low-temperature vibrational spectra but exhibits instability or unphysical behavior under thermal sampling would therefore have limited predictive utility. It is essential to demonstrate that the same potential behaves sensibly in the regime from which macroscopic transport and structural properties are derived.

While both generations of MLIP reproduce the vibrational spectra with comparable accuracy, the two models differ in how they incorporate system-specific training data. The first-generation model was obtained through conventional fine-tuning of the MACE–MP–0 foundation model, in which the network weights are directly updated to reproduce the targeted training configurations. Although this approach is technically straightforward and lowers the barrier to constructing MLIPs, it can in principle lead to *catastrophic forgetting*, whereby aspects of the broader chemical knowledge encoded in the pretrained model are overwritten during fine-tuning. This limitation may become important when the model is used in molecular dynamics simulations, where the system can explore configurations that lie outside the relatively narrow region of configuration space represented in the fine-tuning data set.

By contrast, second-generation foundation models are trained on a much larger data set of approximately 10^8^ atomic configurations and employ a multihead architecture designed to mitigate catastrophic forgetting. This framework allows system-specific refinement while preserving the broader representation of chemical environments learned during pretraining, providing a more robust description when exploring configurations away from the harmonic neighborhood of the training structures. For this reason, the finite-temperature molecular dynamics simulations described below were performed using the second-generation MLIP.

The MD was performed at 300 K in a 1152-atom supercell (Figure 3) using LAMMPS and the MLIAP package.^34^ Over nanosecond time scales, the crystal structure remains intact with no evidence of drift or incipient instability. The mean-squared displacements of all atomic species plateau at physically reasonable values, indicating bounded vibrational and librational motion rather than structural disorder. The components of the stress tensor exhibit stationary fluctuations about zero applied pressure, reflecting mechanical stability of the condensed phase. In equilibrium statistical mechanics, stress autocorrelation functions are directly related to shear viscosity and mechanical response via Green–Kubo formalisms, so the absence of systematic drift or anomalous variance indicates a mechanically coherent description under thermal sampling. Radial distribution functions computed over early and late trajectory windows are indistinguishable, confirming preservation of local bonding environments and intermolecular packing. Because radial distribution functions describe the pair correlations that contribute to diffraction observables, the absence of drift in *g*(*r*) provides a direct structural check on the stability of the simulated ensemble. The potential energy likewise displays bounded, stationary fluctuations about a well-defined mean. The plotted quantity is the potential-energy component monitored during the MLIP MD trajectory; the formal heat-capacity fluctuation relation is written in terms of the total/internal energy. We therefore use the potential-energy trace here as a practical stationarity diagnostic, rather than as a direct calculation of heat capacity.

**Figure 3:**
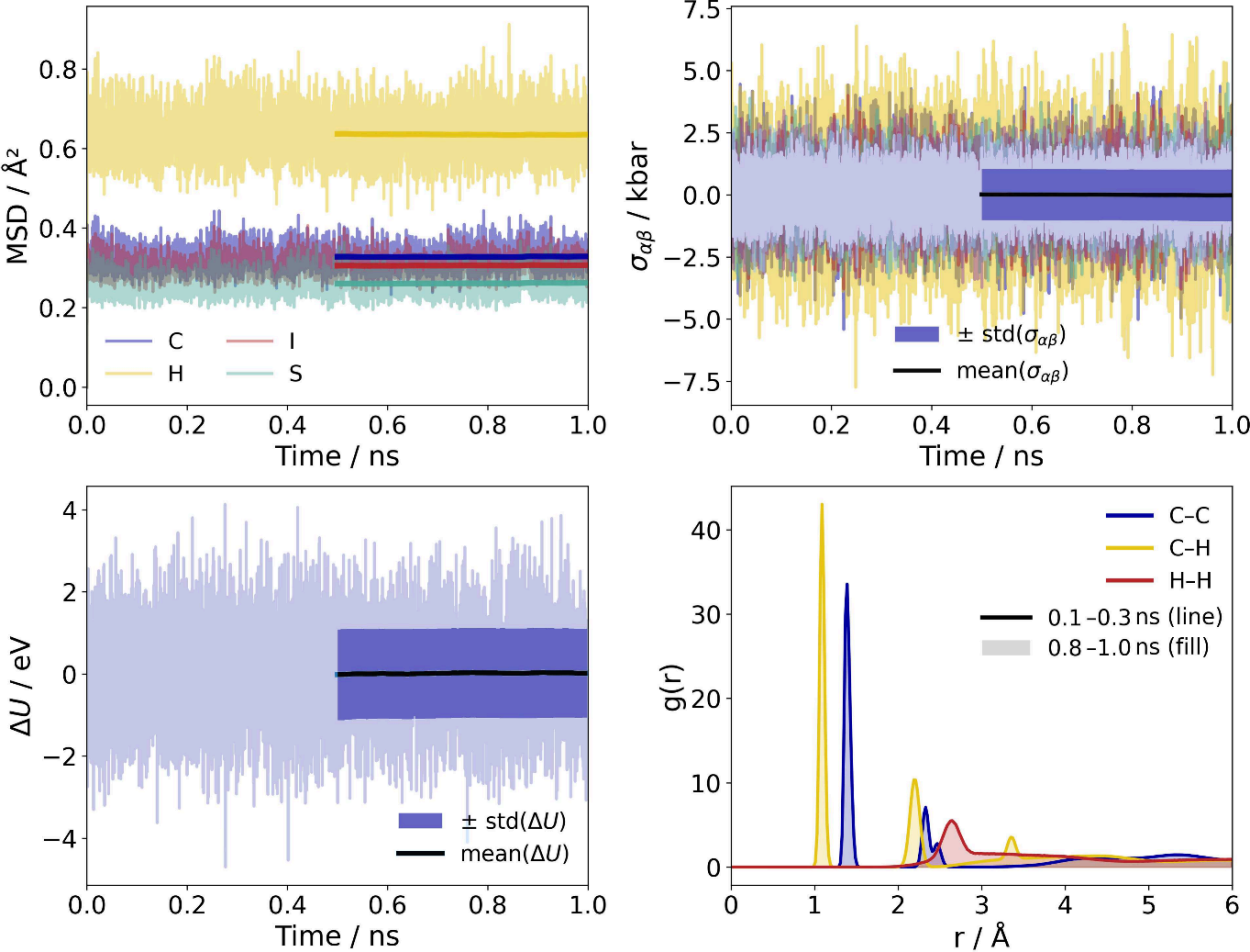
Validation of the 2nd Gen. MLIP (FT-MH-1-PBE-D3) simulation at 300 K. Top left: The mean-squared displacement of each atomic component. Top right: Fluctuations of the components of the stress tensor. Bottom left: Fluctuation of the potential energy. 1000-point running averages are shown for each plot as dark lines, and standard deviations are shown as shaded windows as a guide for the eye. Bottom right: Radial distribution functions for C–C, H–H, and C–H, sampled between 0.1–0.3 ns (solid lines) and between 0.8–1.0 ns (shaded plots).

These results do not constitute direct experimental validation at 300 K; rather, they demonstrate that the microscopic quantities from which macroscopic transport, mechanical, and thermodynamic properties are derived behave sensibly under finite-temperature sampling. The agreement with pressure-dependent INS is therefore embedded within a thermodynamically stable and mechanically consistent description of the system, supporting the use of the MLIP for predictive simulation of dynamical and transport phenomena in molecular materials.

As a final point, we highlight that the refinement protocol appears to be surprisingly robust, with the additional fine-tuned MLIP from R2SCAN-D4 DFT data also producing accurate comparison to experimental phonons (See Supporting Information). This combined with the fact we used three different short-range foundation models (mace-mp-0b3, mace-omat-0 ^26^ and mh-1 ^29^), furthers this case. We also tested the very recently developed electrostatic architectures via MACE-POLAR-1-L,^35^ which despite inclusion of electrostatic long-range forces, appears to still need fine-tuning to describe experiment. Details of these additional calculations are provided in the Supporting Information; however, the central conclusion is that the ultimate predictive accuracy remains governed by the quality of the underlying exchange–correlation functional.

In summary, pressure-dependent INS provides an experimentally grounded route for validating MLIPs under a controlled thermodynamic perturbation. By compressing crystalline 2,5-diiodothiophene while retaining the sharp spectral resolution of 10 K neutron spectroscopy, we probe changes in intermolecular separations, packing geometry, and vibrational force constants within the same molecular crystal. The fine-tuned MACE-based models reproduce the measured spectra at atmospheric pressure and 1.5 GPa, capturing both steric-driven blue shifts and the red shift near 453 cm^–1^ associated with pressure-modified intermolecular interactions. This agreement shows that the learned potential-energy surface captures not only equilibrium vibrational structure, but also its response to compression.

The finite-temperature molecular dynamics simulations provide a complementary stability test beyond the harmonic regime. For the selected second-generation model, the mean-squared displacements, stress components, potential energy, and radial distribution functions remain bounded and stationary over nanosecond sampling at 300 K. These simulations do not constitute direct experimental validation at finite temperature, but they show that the model behaves sensibly in the regime from which transport, thermodynamic, and structural observables would be extracted.

High-pressure INS therefore provides a practical experimental framework for assessing MLIP transferability in molecular materials. When combined with finite-temperature stability checks, this approach can help identify potentials that are not merely interpolative reproductions of training data, but useful models of pressure-dependent structure–dynamics relationships and molecular force landscapes.

## Supplementary Material



## Data Availability

Data are available at the following repository https://data-collections.psdi.ac.uk/records/exkx8-z1y71.

## Notes

Experimental methods; data reduction and background subtraction; density-functional theory calculations; machine-learned interatomic potential training protocol and computational cost; structural comparisons; harmonic lattice-dynamics and mode assignments; AbINS simulations; finite-temperature molecular dynamics validation; equation-of-state analysis (PDF)
