# Supplementary material for: High-Pressure Inelastic Neutron Spectroscopy: Experimental Validation of Machine-Learned Interatomic Potential Energy Landscapes
Source: J Phys Chem Lett. 2026 Jun 5;17(24):6596–601. doi: 10.1021/acs.jpclett.6c00720 (PMC13288705; doi:10.1021/acs.jpclett.6c00720)
Supplement: Supplementary file 1 [file jz6c00720_si_001.pdf]

High-Pressure Inelastic Neutron Spectroscopy: Experimental validation of Machine-Learned Interatomic Potential energy landscapes

Supporting Information

Jeff Armstrong*[1,2], Adam Jackson[3], and Alin Elena†[4]

[1]ISIS Neutron and Muon Source, Science and Technology Facilities Council, UK Research and Innovation, Rutherford Appleton Laboratory, Harwell Campus, Didcot, OX11 0QX, UK
[2]Department of Chemistry, University of Bath, Claverton Down, Bath, BA2 7AY, UK
[3]Scientific Computing Department, Science and Technology Facilities Council, UK Research and Innovation, Rutherford Appleton Laboratory, Harwell Campus, Didcot, OX11 0QX, UK
[4]Scientific Computing Department, Science and Technology Facilities Council, UK Research and Innovation, Daresbury Laboratory, Keckwick Lane, Daresbury, WA4 4AD, UK

Contents

S1 Experimental methods **2**

S2 Data reduction and background subtraction **2**

S3 Density-functional theory calculations **4**

S4 MLIP training protocol and dataset construction **4**

S5 Structural data comparison **7**

S6 Harmonic lattice dynamics and mode assignment **8**

S7 INS simulations with ABINS **10**

S8 Finite-temperature molecular dynamics validation **10**

S9 Equation-of-state analysis **11**

*Corresponding author: jeff.armstrong@stfc.ac.uk
†Corresponding author: alin-marin.elena@stfc.ac.uk
Jeff Armstrong ORCID: 0000-0002-8326-3097
Adam Jackson ORCID: 0000-0001-5272-6530
Alin Elena ORCID: 0000-0002-7013-6670

S1 Experimental methods

S1.1 Sample and measurement conditions

All inelastic neutron scattering (INS) measurements were performed on the TOSCA inelastic neutron spectrometer at the ISIS Neutron and Muon Source.[1] Ambient-pressure spectra were collected at 10 K using a 2 g sample of crystalline 2,5-diiodothiophene loaded into a standard aluminium sample can. High-pressure spectra were collected at 10 K at 1.5 GPa using a 200 mg sample loaded into a low-background NiCrAl clamp cell. The use of cryogenic temperature suppresses large-amplitude molecular motion, thus minimizing the Debye-Waller peak suppression and improving the quantitative comparison with harmonic lattice-dynamical calculations.

S1.2 High-pressure clamp cell and masking

The 1.5 GPa measurements were carried out in a NiCrAl alloy clamp cell designed to minimise parasitic neutron scattering while retaining mechanical robustness at gigapascal pressures.[2] To further suppress scattering from the bulk body of the clamp assembly, a cadmium mask was employed to restrict the illuminated volume to the sample region. In practice, the residual cell background depends weakly on small differences in mask positioning, sample centreing, and multiple scattering contributions.

S1.3 Pressure generation and reproducibility

Pressure was applied ex situ by mechanical loading of the clamp cell with 3 tonnes of weights. The reported pressure corresponds to the nominal applied load for the calibrated clamp geometry when cooled to 10 K.

S2 Data reduction and background subtraction

S2.1 Empty-cell subtraction

For the high-pressure measurement, cell-background subtraction was performed using an independently measured empty-cell spectrum acquired under nominally identical instrument settings. Because the 1.5 GPa dataset has a substantially lower sample mass and additional attenuation through the pressure cell, the high-pressure spectra exhibit an elevated statistical noise floor relative to the ambient-pressure dataset. Nevertheless, peak positions and relative pressure-dependent shifts remain well-resolved across the 0–1200 cm^{-1} range.

S2.2 Practical considerations and uncertainty

Residual artefacts after subtraction can arise from:

- small differences in the effective illuminated volume (mask and cell positioning),
- multiple scattering contributions that depend on sample loading,

Figure S1 shows a comparison of the cell signal and sample signals before and after subtraction, to give the reader an idea of the relative intensities. A zoomed in plot of the experimental subtracted spectra is shown in Figure S2.

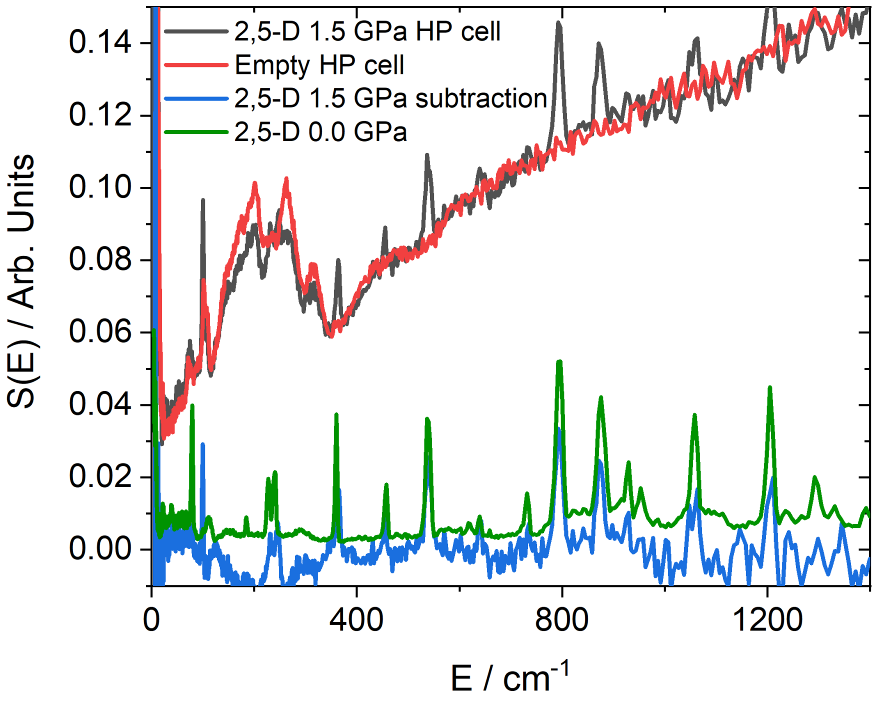

Figure S1: Subtracted TOSCA INS spectra of 2,5-D at ambient pressure (green) is compared to the 1.5 GPa measurement (blue). Unsubtracted data at 1.5 GPa (black) and the accompanying empty pressure cell (red) are included to give the reader insight into the background.

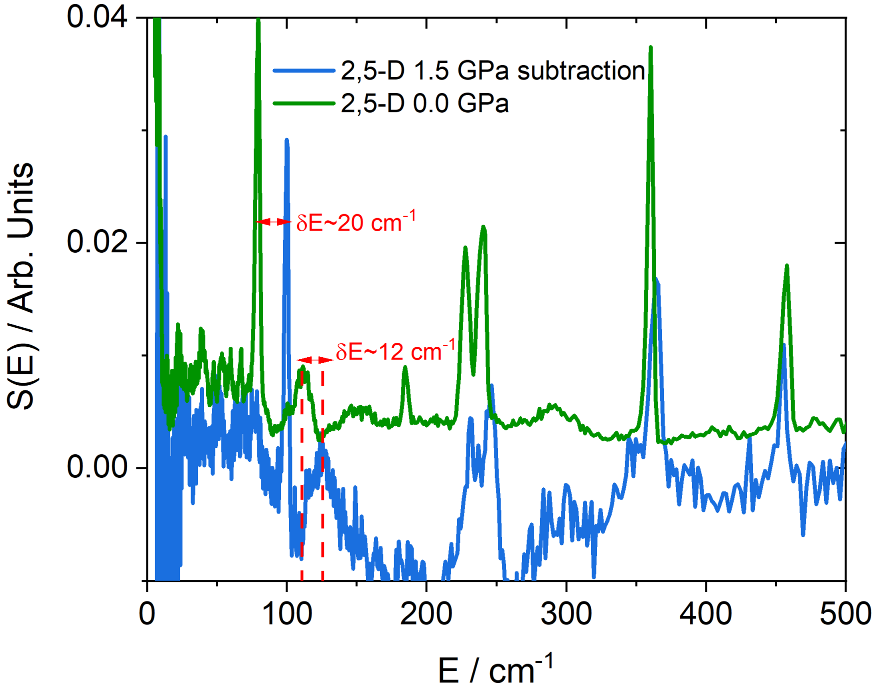

Figure S2: An energy expanded plot of the two fully subtracted spectra; ambient pressure in green, 1.5 GPa in blue.

S3 Density-functional theory calculations

S3.1 Electronic structure parameters

Density-functional theory (DFT) calculations were performed using the projector-augmented wave (PAW) method as implemented in VASP.[3,4] Structures were optimised at the target pressures by applying an external stress and relaxing both lattice parameters and atomic positions.

A plane-wave cutoff energy of 520 eV was used for calculations with the PBE exchange-correlation (XC) functional and Grimme D3 correction including Becke–Johnson damping.[5,6] The PBE-D3(BJ) calculations used the internal VASP implementation with `IVDW = 12`, for which the Axilrod–Teller–Muto three-body term is not included. (The corresponding MACE models are given a "-PBE-D3" suffix.) Reciprocal space was sampled automatically with a target spacing of $0.2\,\text{Å}^{-1}$, shifted away from the Γ-point. Electronic self-consistency was converged to 1×10^{-7} eV per calculation cell or less, using Gaussian smearing of 0.01 eV. The cutoff energy and PAW datasets were selected to remain consistent with the reference setup used for the MACE-MP-0 foundation model training data, ensuring compatibility between the baseline electronic-structure description and the MLIP fine-tuning workflow.[7]

For the R2SCAN-D4 dataset slightly different parameters were used (including a 680 eV plane-wave cutoff and **k**-point sampling offset to Γ) as documented for the MatPES dataset,[8] to which a compatible D4 correction was added.[9]

S3.2 Functional sensitivity

To assess sensitivity to the underlying electronic-structure description, additional models were fine tuned at different levels of theory and fine tuning methodology, see details in the next section of the models. Calculations were performed using the FT-MH-1-PBE-D3, MACE-POLAR-1-L, and FT-R2SCAN-D4, while keeping all other numerical settings fixed. This provides a controlled basis for interpreting differences between refined machine-learning interatomic potential (MLIP)s as differences in the reference potential-energy surface, rather than differences in convergence settings. Figure S3 shows the various MLIP spectra from our study. Figure S3 also includes the unfine-tuned MACE-POLAR-1-L foundation model as a representative test of out-of-the-box foundation-model performance. Despite including long-range electrostatics and being trained on the OMol25 dataset at the ωB97M-V/def2-TZVPD level of theory, MACE-POLAR-1-L does not reproduce the experimental spectra as well as the short-range models after system-specific fine-tuning. This comparison illustrates that, for the present molecular crystal, foundation-model transferability is improved substantially by targeted fine-tuning to the relevant condensed-phase configurations.

S4 MLIP training protocol and dataset construction

S4.1 Overview

MLIP calculations were performed using the MACE architecture with GPU acceleration via PyTorch.[10,11] A system-specific potential was obtained by fine-tuning the MACE-MP-0b3 "foundation model" on targeted DFT data.[7] The guiding objective of the refinement protocol is twofold: (i) reproduce the harmonic vibrational response at both zero pressure and 1.5 GPa, and (ii) ensure stability and physically sensible behaviour under finite-temperature sampling.

S4.2 Training configurations and fine-tuning

Training configurations were generated via four techniques.

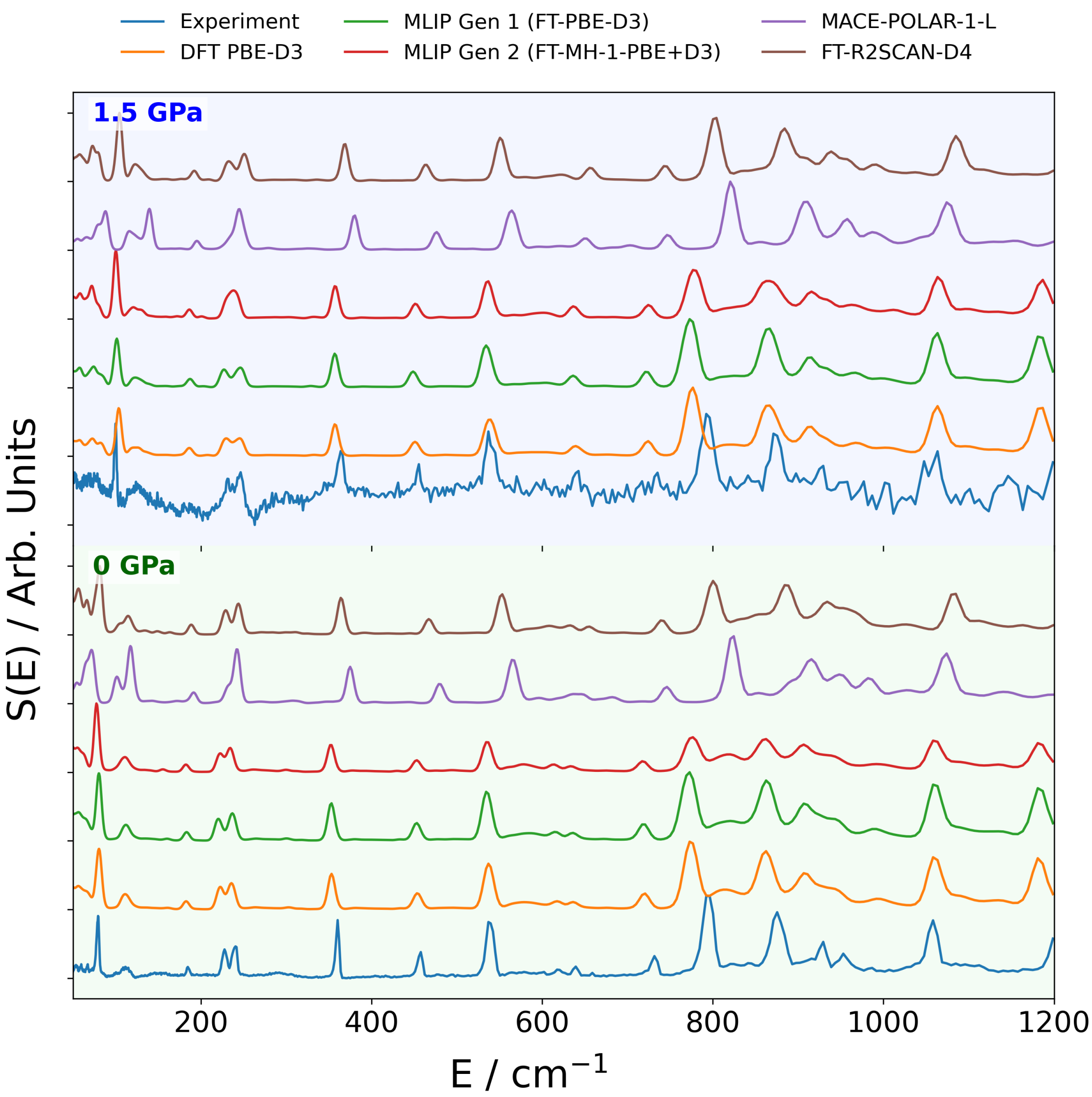

Figure S3: Vibrational spectra $S(E)$ for experiment, DFT, finetuned MLIPs and newly released polarisable molecular foundation model MACE-POLAR-1-L. Data for the two pressures 0 GPa and 1.5 GPa are shown in the lower green panel and upper blue panel respectively.

Geometry optimisation of the the perfect crystal using DFT at PBE-D3(BJ) level, then from the resulting trajectory only frames that differ by more than 50 meV were kept in the training set.

Volume Scans, using equation of state approach we compressed the initial crystal by 10% of its volume and relaxed to the same amount in 11 steps. For all these single point calculations were performed and data added in the training set.

Molecular dynamics: NPT trajectories were run under a Nosé–Hoover thermostat at 300 K on a unit-cell using JANUS-CORE, with a length of max 1 ns saving configurations with a 1 ps frequency, using different pressures, 0, 0.5, 1.0 and 1.5, 2.0 GPa. Each such generated trajectory has MACE descriptors computed on each frame and further point sampling is used to sample a number of frames (max 100) in this reduced space. The selected frames are split in and 8:1:1 ratio between training, validation and testing sets.

Non-diagonal supercells: After five initial training cycles, additional 288-atom configurations were generated using alternative supercell matrices originally developed for efficient phonon sampling and recently repurposed for MLIP training.[12,13] The supercell matrices

$$\begin{pmatrix} 2 & 1 & 0 \\ 2 & -1 & 0 \\ 0 & 0 & 1 \end{pmatrix}, \quad \begin{pmatrix} 4 & 0 & 0 \\ 0 & 1 & 0 \\ 2 & 0 & -1 \end{pmatrix}, \quad \begin{pmatrix} 2 & -1 & 0 \\ -2 & 0 & 1 \\ 2 & 1 & 0 \end{pmatrix}$$

were used to construct simulation cells. Molecular dynamics was run in the NPT ensemble using ASE at 300 K and 0 GPa for 20 000 steps with a 1 fs time step. Snapshots were drawn randomly from the trajectories after a thermalisation period, with the random seeds selected for a satisfactory distribution of energies and lattice vectors. They were labelled with DFT energies, forces, and stresses, and incorporated into subsequent training iterations. These samples were intended to identify/address any finite-size effects related to the consistent use of particular periodic boundaries. At the end of the process, we have 1412 structures in the training set, 176 in test and 176 in validation set.

The first fine-tuned model involved naive fine-tuning against mace_mp_0b3, using a loss stress and learning rate of 0.005 and an ema_decay of 0.995, a batch size of 8 and 2000 epochs. Fittings involved an energy RMSE of 10.0 meV atom^{-1}, 8.4 meVÅ^{-1} for forces, and 0.7 meVÅ^{-3} for stresses. We used an A100 NVIDIA GPU for the fine tuning with a runtime of 20s per epoch. While this was the preferred method at the time of finetuning, naive fine-tuning is associated with the risk of "catastrophic forgetting"[14]. This is the first generation model in the main text (i.e. **FT-PBE-D3**). No observed issues were assigned to catastrophic forgetting during this work.

For fine tuning against R2SCAN-D4 DFT we selected randomly 1068 of the existing structures for training and 133 for the validation and test sets, and recomputed the energy, forces and stress at the new level of theory. We performed multihead fine-tuning against mace_omat-0, using loss universal and learning rate of 0.0001 and an ema_decay of 0.99999, a batch size of 2 and 10 epochs. The replay dataset used was a combination of Materials project and OMAT datasets, as available on MACE website, and we used the original DFT labels. The fitting results were 1.05 meV atom^{-1} RMSE of energy, 26.59 meVÅ^{-1} RMSE of forces, and 0.48 meVÅ^{-3} RMSE of stresses. We used 4 A100 NVIDIA GPUs for the fine tuning with a runtime of 1h and 18 minutes per epoch. The resulting model is called **FT-R2SCAN-D4**.

The third finetuning strategy used the same structures as in R2SCAN-D4 case but at PBE-D3(BJ) level of theory. We performed multihead fine-tuning against mh-1 model, head omat_pbe, using loss stress and learning rate of 0.0001 and an ema_decay of 0.99999, a batch size of 2 and 10 epochs, for second stage fine tuning use reduced learning rate by one order of magnitude. The replay dataset used Materials project dataset, from which we selected 30000 samples that contain combinations of elements with atomic numbers 1, 6, 16, 53, and we used pseudolabels computed with mh-1 model, head omat_pbe for these structures. Fitting results were 0.94 meVatom^{-1} RMSE of energy, 22.24 meVÅ^{-1} RMSE of forces, and 0.30 meVÅ^{-3} RMSE of stresses. The runtime on 4 A100 NVIDIA GPUs was 56 minutes per epoch. This model is

named **FT-MH-1-PBE-D3** and was used to produce all the data for molecular dynamics simulations in the main paper; it is also compared in Figure 1 of the main text to the experimental phonons and FT-PBE-D3.

S4.3 Computational cost of DFT data generation, fine-tuning, and phonon prediction

The construction of a fine-tuned MLIP involves a non-negligible upfront computational cost associated with generating DFT energies, forces, and stresses for the training configurations, followed by GPU-based model refinement. This cost should therefore be distinguished from the much lower cost of subsequent geometry optimisation, phonon prediction, and finite-temperature sampling with the trained potential.

For the present system, the first-generation PBE-D3(BJ) training workflow used a final dataset containing 1412 training structures, 176 validation structures, and 176 test structures. The multi-head PBE-D3(BJ) and R2SCAN-D4 fine-tuning workflows used 1068 training structures, 133 validation structures, and 133 test structures. A representative DFT single-point calculation required approximately 25 min using four 32-core AMD nodes on the SCARF high-performance computing facility. The first-generation fine-tuned model was trained for 2000 epochs and required approximately 11 h wall time on NVIDIA A100 GPU resources. The second-generation multi-head PBE-D3(BJ) model was trained for 10 epochs and required approximately 7 h 38 min wall time on NVIDIA A100 GPU resources.

Once the potential was trained, the cost of phonon prediction was substantially reduced. For the $2\times2\times4$ supercell used for the MLIP phonon calculations, geometry optimisation to forces below 10^{-6} eV Å^{-1} followed by phonon band and density-of-states calculations required 3 min 23 s on a single NVIDIA A100 GPU. The same workflow could also be run on a desktop CPU, requiring 5 min 19 s.

These timings show that the computational benefit of the MLIP workflow is not best understood as replacing a single DFT phonon calculation. Instead, the cost of generating the DFT training data and fine-tuning the model is paid once, after which the resulting potential can be reused for repeated geometry optimisations, phonon calculations, pressure scans, larger supercells, and finite-temperature molecular dynamics. The pressure-dependent INS comparison in the main text therefore serves as an experimental validation step for a reusable potential-energy surface, rather than simply as a lower-cost route to one harmonic spectrum.

A systematic learning-curve analysis, in which the number and type of DFT training configurations are varied independently and the resulting phonon spectra, pressure shifts, and finite-temperature stability are re-evaluated, is beyond the scope of the present work. Here, the training set was designed pragmatically to span the relevant structural perturbations for this material, including equilibrium configurations, volume changes, pressure-optimised structures, finite-temperature distortions, and non-diagonal supercell configurations. The resulting models are therefore assessed by their ability to reproduce the DFT reference response, the pressure-dependent INS spectra, and stable finite-temperature dynamics, rather than by identifying the minimum possible training set size.

S5 Structural data comparison

Table S1 compares the relaxed lattice parameters obtained from DFT, unfine-tuned foundation models, and fine-tuned MLIPs at atmospheric pressure and 1.5 GPa.

Table S1: Lattice parameters at different pressures and simulation methods.

Pressure: 0 GPa

Method	a [Å]	b [Å]	c [Å]	α [°]	β [°]	γ [°]
exp	14.9443(9)	18.2941(15)	5.2541(3)	90.0	90.0	90.0
DFT (PBE-D3(BJ))	14.895	18.123	5.136	90.0	90.0	90.0
MACE_MP-0b3-D3*	14.694	17.988	5.166	90.0	90.0	90.0
MACE_MP-0b3-D3	12.705	18.236	5.702	90.0	90.0	90.0
MACE_MP-OMAT-0-D3	15.776	17.199	5.543	90.0	90.0	90.0
MACE-MH-1 OMAT_PBE-D3	13.192	18.141	5.825	90.0	90.0	90.0
MACE-POLAR-1-L	12.729	18.240	5.921	90.0	90.0	90.0
FT-PBE-D3	14.915	18.114	5.129	90.0	90.0	90.0
FT-R2SCAN-D4	14.745	18.382	5.301	90.0	90.0	90.0
FT-MH-1-PBE-D3	14.606	18.165	5.251	90.0	90.0	90.0
FT-MH-1-PBE-D3*	14.791	18.107	5.200	90.0	90.0	90.0

Pressure: 1.5 GPa

Method	a [Å]	b [Å]	c [Å]	α [°]	β [°]	γ [°]
exp	—	—	—	—	—	—
DFT (PBE-D3(BJ))	14.310	17.322	5.008	90.0	90.0	90.0
MACE_MP-0b3-D3*	14.088	17.246	4.953	90.0	90.0	90.0
MACE_MP-0b3-D3	11.872	17.329	5.723	90.0	90.0	90.0
MACE_MP-OMAT-0-D3	13.454	17.266	5.580	90.0	90.0	90.0
MACE-MH-1 OMAT_PBE-D3	12.817	17.311	5.607	90.0	90.0	90.0
MACE-POLAR-1-L	12.385	17.494	5.762	90.0	90.0	90.0
FT-PBE-D3	14.358	17.335	4.989	90.0	90.0	90.0
FT-R2SCAN-D4	14.121	17.499	5.189	90.0	90.0	90.0
FT-MH-1-PBE-D3	14.060	17.348	5.112	90.0	90.0	90.0
FT-MH-1-PBE-D3*	14.265	17.463	5.015	90.0	90.0	90.0

Only cell vectors and positions were optimised; angles were fixed.

S6 Harmonic lattice dynamics and mode assignment

Phonon force constants were obtained from MLIPs using finite displacements with PHONOPY.[15] Peak assignments were obtained from the Γ-point MLIP phonon eigenvectors at 0 GPa, guided by atomic displacement character and visual inspection of eigenmodes. The dominant pressure response below 500 cm^{-1} arises from modes that modulate intermolecular contacts and steric repulsion within the herringbone-like packing motif, while the anomalous red shift near 453 cm^{-1} corresponds to an out-of-plane deformation of the thiophene ring (C–S torsional character).

In addition to gamma point phonon calculations, we also calculated phonon dispersion curves to compare with DFT. This provides a particularly fine test of the reproduction of the dynamics, as the dispersion patterns are particularly fine features. Figure S4 shows the band diagram of this comparison.

Table S2: Peak assignments from 0 GPa MLIP phonon calculations.

Peak position / cm^{-1}	Description
80	Libration
105–116	C–I in-phase out-of-plane and in-plane bend
182	C–I symmetric stretch
220	C–I out-of-phase out-of-plane bend
237	C–I out-of-phase in-plane bend
353	C–I antisymmetric stretch
453	Out-of-plane ring deformation (C–S torsion)

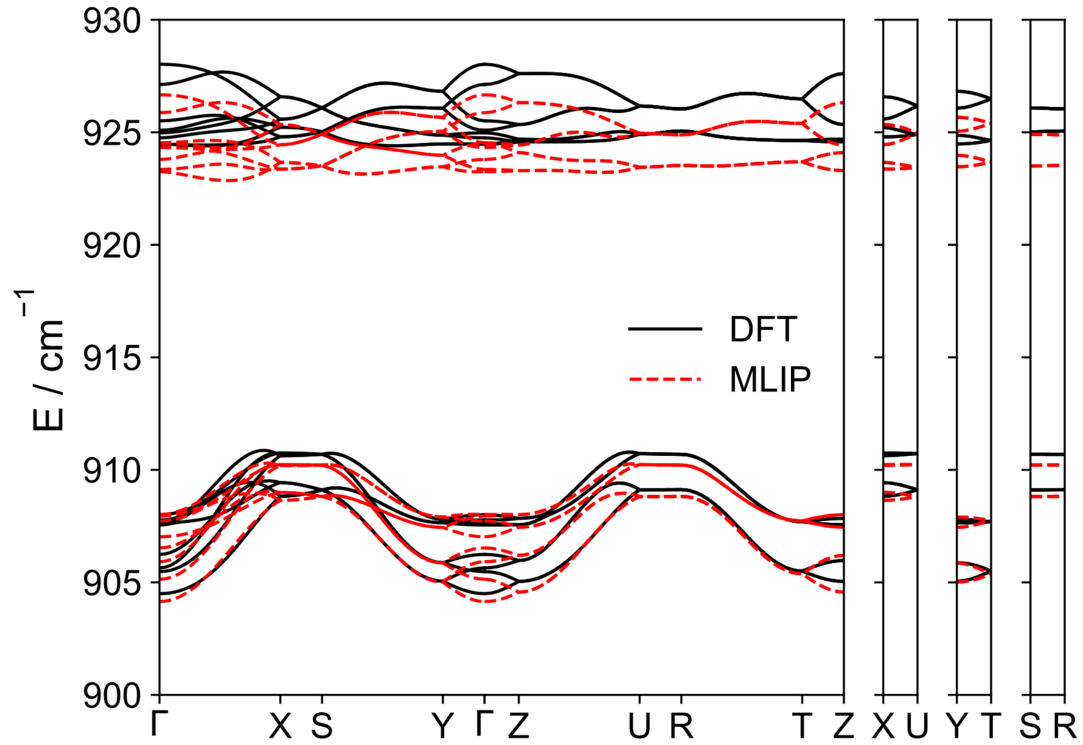

Figure S4: A direct comparison of the DFT and MLIP phonon bands. We have zoomed into the deformation region to allow the comparison of the subtle dispersion pattern in this region.

S7 INS simulations with ABINS

INS spectra from simulated data were produced using the ABINS code distributed as part of MANTID[16,17]. Phonon force constants were obtained from the MLIPs using PHONOPY via the JANUS-CORE command-line tools. Force constants were converted into phonon frequencies and eigenvectors, which were then used by ABINS to compute neutron-weighted vibrational spectra appropriate for direct comparison with TOSCA measurements. ABINS employs a density of states (DOS)-like incoherent approximation to the INS spectrum, computing intensities along the kinematically constrained ω–Q trajectories of each TOSCA detector bank, and applies an energy-dependent Gaussian resolution function. The one-phonon spectrum and second-order combination modes were enumerated within the "almost-isotropic approximation", and a fully isotropic convolution approximation was used to include higher quantum orders (3–10). These higher-order contributions are important for reproducing the elevated background at higher energies and for ensuring consistent intensity scaling across the full 0–1200 cm^{-1} range.

S8 Finite-temperature molecular dynamics validation

S8.1 Simulation protocol

To assess thermodynamic stability beyond the harmonic limit, the final MLIP was validated using molecular dynamics (MD) simulations at 300 K under periodic boundary conditions, employing a 1152-atom supercell. The system was equilibrated in the NPT ensemble for 500 ps, followed by a production run in the NVT ensemble of 1 ns to collect statistics. Stability was assessed through complementary structural, thermodynamic, and dynamical metrics, including the mean-squared displacement (MSD), stress tensor fluctuations, potential energy stationarity, and radial distribution function (RDF) invariance over time.

S8.2 Metrics and interpretation

The integrity of the molecular geometry and packing motif was monitored using intra- and intermolecular RDFs $g(r)$ for C–C, C–H, and H–H pairs. To explicitly test for temporal drift, $g(r)$ was evaluated in an early (0.1–0.3 ns) and late (0.8–1.0 ns) window of the NVT trajectory; indistinguishable distributions indicate preservation of bonding environments and intermolecular packing. Stress tensor components exhibit stationary fluctuations without systematic drift, consistent with mechanical stability of the condensed phase. Potential energy fluctuations remain bounded around a well-defined mean, indicating absence of slow relaxation or instability. Finally, MSDs for all atomic species plateau at physically reasonable values, consistent with bounded vibrational and librational motion and the absence of diffusive runaway behaviour under these conditions.

Table S3: Summary of finite-temperature MD validation conditions (reported as used in the main manuscript).

Quantity	Value
Supercell size	1152 atoms
Temperature	300 K
Equilibration	NPT, 500 ps
Production	NVT, 1 ns (main text)
Time step	1 fs
Key observables	MSD, stress tensor, ΔU, RDF invariance

S9 Equation-of-state analysis

Additional calculations were performed on the generated MLIP to assess how lattice parameters and lattice energy evolves as a function of pressure. Energy–volume curves were produced using JANUS-CORE: at each point the experimental structure is scaled then the lattice parameters and geometry optimised with the MLIP under a fixed-volume constraint. The Birch–Murnaghan equation of state was fitted to the resulting data and used to obtain a hydrostatic pressure estimate at each point. The results are contained in Figure S5. Note that the visualisation and discussion in the main manuscript refer to Cartesian directions (x, y, z); the lattice vectors a, b, c are orthogonal and aligned with x, z and y respectively.

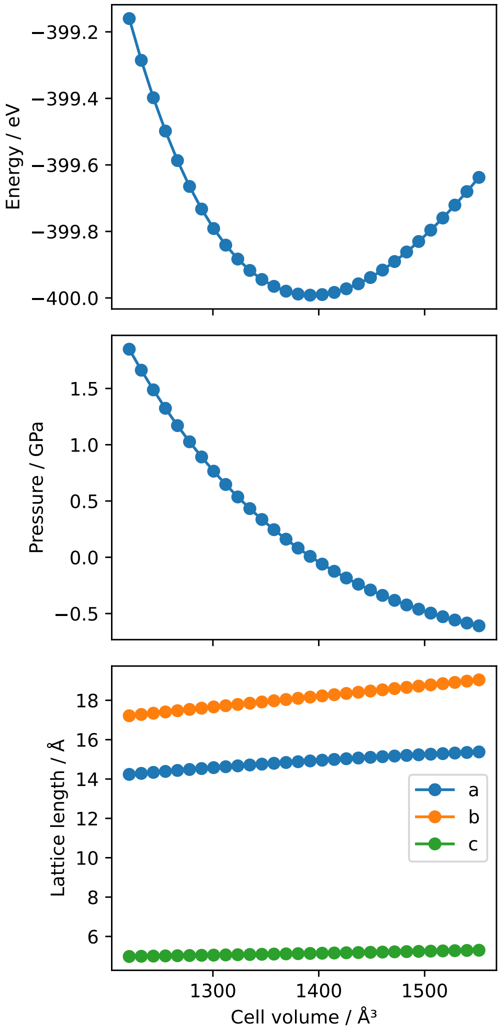

Figure S5: Lattice properties as a function of cell volume. Top: lattice energy, middle:pressure, bottom: individual lattice vector length.

At each volume the rings were identified and isolated using neighbour-lists in ASE, and their out-of-plane (normal) vectors identified by principle-component analysis in scikit-learn.[18,19] The vectors were split into two groups (corresponding to each side of a "herringbone" row of molecules in 2,5-diiodothiophene) and a representative angle calculated between their averages. The evolution of this angle is plotted against the corresponding EOS pressure in Figure S6.

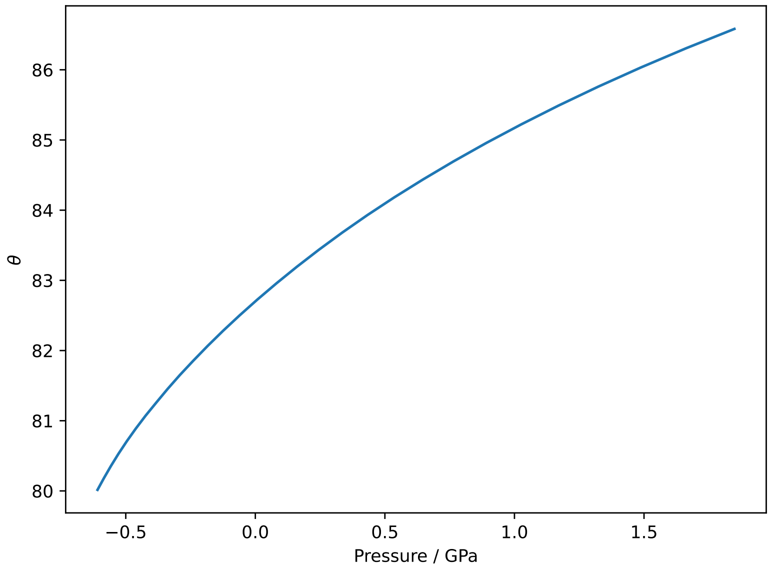

Figure S6: Angle between the ring normals on each side of the "herringbone" arrangement of molecules in 2,5-diidothiophene as a function of pressure. This was calculated using structures and Birch–Murnaghan equation of state from MLIP energy-volume curve (Figure S5).

References

(1) R. S. Pinna, S. Rudić, S. F. Parker, J. Armstrong, M. Zanetti, G. Škoro, S. P. Waller, D. Zacek, C. A. Smith, M. J. Capstick, D. J. McPhail, D. E. Pooley, G. D. Howells, G. Gorini and F. Fernandez-Alonso, *Nuclear Instruments and Methods in Physics Research Section A: Accelerators, Spectrometers, Detectors and Associated Equipment*, 2018, **896**, 68–74.

(2) J. Armstrong, X. Wang and F. Fernandez-Alonso, *Nuclear Instruments and Methods in Physics Research Section A: Accelerators, Spectrometers, Detectors and Associated Equipment*, 2022, **1039**, 167097.

(3) G. Kresse and J. Furthmüller, *Physical Review B*, 1996, **54**, 11169–11186.

(4) G. Kresse and D. Joubert, *Physical Review B*, 1999, **59**, 1758–1775.

(5) J. P. Perdew, K. Burke and M. Ernzerhof, *Physical Review Letters*, 1996, **77**, 3865–3868.

(6) S. Grimme, S. Ehrlich and L. Goerigk, *Journal of Computational Chemistry*, 2011, **32**, 1456–1465.

(7) I. Batatia, P. Benner, Y. Chiang, A. M. Elena, D. P. Kovács, J. Riebesell, X. R. Advincula, M. Asta, M. Avaylon, W. J. Baldwin, F. Berger, N. Bernstein, A. Bhowmik, F. Bigi, S. M. Blau, V. Cărare, M. Ceriotti, S. Chong, J. P. Darby, S. De, F. Della Pia, V. L. Deringer, R. Elijošius, Z. El-Machachi, E. Fako, F. Falcioni, A. C. Ferrari, J. L. A. Gardner, M. J. Gawkowski, A. Genreith-Schriever, J. George, R. E. A. Goodall, J. Grandel, C. P. Grey, P. Grigorev, S. Han, W. Handley, H. H. Heenen, K. Hermansson, C. H. Ho, S. Hofmann, C. Holm, J. Jaafar, K. S. Jakob, H. Jung, V. Kapil, A. D. Kaplan, N. Karimitari, J. R. Kermode, P. Kourtis, N. Kroupa, J. Kullgren, M. C. Kuner, D. Kuryla, G. Liepuoniute, C. Lin, J. T. Margraf, I.-B. Magdău, A. Michaelides, J. H. Moore, A. A. Naik, S. P. Niblett, S. W. Norwood, N. O'Neill, C. Ortner, K. A. Persson, K. Reuter, A. S. Rosen, L. A. M. Rosset, L. L. Schaaf, C. Schran, B. X. Shi, E. Sivonxay, T. K. Stenczel, C. Sutton, V. Svahn, T. D. Swinburne, J. Tilly, C. van der Oord, S. Vargas, E. Varga-Umbrich, T. Vegge, M. Vondrák, Y. Wang, W. C. Witt, T. Wolf, F. Zills and G. Csányi, *The Journal of Chemical Physics*, 2025, **163**, 184110.

(8) A. D. Kaplan, R. Liu, J. Qi, T. W. Ko, B. Deng, J. Riebesell, G. Ceder, K. A. Persson and S. P. Ong, *A foundational potential energy surface dataset for materials*, arXiv:2503.04070, 2025.

(9) S. Ehlert, U. Huniar, J. Ning, J. W. Furness, J. Sun, A. D. Kaplan, J. P. Perdew and J. G. Brandenburg, *The Journal of Chemical Physics*, 2021, **154**, 061101.

(10) I. Batatia, D. P. Kovács, G. N. C. Simm, C. Ortner and G. Csányi, Advances in Neural Information Processing Systems, 2022, vol. 35, pp. 11423–11436.

(11) D. P. Kovács, I. Batatia, E. S. Arany and G. Csányi, *The Journal of Chemical Physics*, 2023, **159**, 044118.

(12) J. H. Lloyd-Williams and B. Monserrat, *Physical Review B*, 2015, **92**, 184301.

(13) C. Allen and A. P. Bartók, *Machine Learning: Science and Technology*, 2022, **3**, 045031.

(14) I. Batatia, C. Lin, J. Hart, E. Kasoar, A. M. Elena, S. W. Norwood, T. Wolf and G. Csányi, *Cross learning between electronic structure theories for unifying molecular, surface, and inorganic crystal foundation force fields*, arXiv:2510.25380, 2025.

(15) A. Togo, *Journal of the Physical Society of Japan*, 2023, **92**, 012001.

(16) K. Dymkowski, S. F. Parker, F. Fernández-Alonso and S. Mukhopadhyay, *Physica B: Condensed Matter*, 2018, **551**, 443–448.

(17) O. Arnold, J. C. Bilheux, J. M. Borreguero, A. Buts, S. I. Campbell, L. Chapon, M. Doucet, N. Draper, R. F. Leal, M. A. Gigg, V. E. Lynch, A. Markvardsen, D. J. Mikkelson, R. L. Mikkelson, R. Miller, K. Palmen, P. Parker, G. Passos, T. G. Perring, P. F. Peterson, S. Ren, M. A. Reuter, A. T. Savici, J. W. Taylor, R. J. Taylor, R. Tolchenov, W. Zhou and J. Zikovsky, *Nuclear Instruments and Methods in Physics Research Section A: Accelerators, Spectrometers, Detectors and Associated Equipment*, 2014, **764**, 156–166.

(18) A. Hjorth Larsen, J. Jørgen Mortensen, J. Blomqvist, I. E. Castelli, R. Christensen, M. Dułak, J. Friis, M. N. Groves, B. Hammer, C. Hargus, E. D. Hermes, P. C. Jennings, P. Bjerre Jensen, J. Kermode, J. R. Kitchin, E. Leonhard Kolsbjerg, J. Kubal, K. Kaasbjerg, S. Lysgaard, J. Bergmann Maronsson, T. Maxson, T. Olsen, L. Pastewka, A. Peterson, C. Rostgaard, J. Schiøtz, O. Schütt, M. Strange, K. S. Thygesen, T. Vegge, L. Vilhelmsen, M. Walter, Z. Zeng and K. W. Jacobsen, *Journal of Physics: Condensed Matter*, 2017, **29**, 273002.

(19) F. Pedregosa, G. Varoquaux, A. Gramfort, V. Michel, B. Thirion, O. Grisel, M. Blondel, P. Prettenhofer, R. Weiss, V. Dubourg, J. Vanderplas, A. Passos, D. Cournapeau, M. Brucher, M. Perrot and E. Duchesnay, *Journal of Machine Learning Research*, 2011, **12**, 2825–2830.
